# Medical Orgone Therapy, a relic of the past or a technique for the future?

**DOI:** 10.1192/j.eurpsy.2023.2172

**Published:** 2023-07-19

**Authors:** A. Foglia

**Affiliations:** Psychiatry, Swiss Board of Psychiatry, Paradiso, Switzerland

## Abstract

**Introduction:**

We introduce and present a psychotherapic technique initiated by Wilhelm Reich (1897-1957) in the 1920’s and thereafter expanded and refined with essential contributions by Elsworth Baker among others. We outline its theory and technical foundations and also provide an epistemologic interpretation of its peculiar body-mind representation based on character- and muscular armoring as a reaction to suffering; armoring of the ocular segment in the etiology of Bleuler’s schizophrenic splitting; and finally the consequences of armoring on the energetic functions as the logical development of Freud’s theory of libido, with significant theoretic, therapeutic and nosologic consequences.

**Objectives:**

The goal of my presentation is to avoid extinction of a valuable technique for future psychiatrists; moreover, to catch the interest of young collegues to a technique that integrates Bleuler’s psychiatric discovery with Freud’s psychoanalytic contribution and demonstrates that the old nosology and theoretical achievements in the psychic field were not as obsolete as today many think.

**Methods:**

Research, book summaries, practical experience

**Results:**

Poster presentation

**Conclusions:**

Poster presentation

**Disclosure of Interest:**

None Declared

